# Determinants of referral for suspected coronary artery disease: a qualitative study based on decision thresholds

**DOI:** 10.1186/s12875-023-02064-y

**Published:** 2023-05-02

**Authors:** Katja Winkler, Navina Gerlach, Norbert Donner-Banzhoff, Anika Berberich, Jutta Jung-Henrich, Kathrin Schlößler

**Affiliations:** 1 Department of General Practice/Family Medicine, University Marburg, Karl-Von-Frisch-Str. 4, 35043 Marburg, Germany; 2 Institute of General Practice and Family Medicine (AM RUB), Ruhr University, Bochum, Germany

**Keywords:** Consultation and Referral, Primary Health Care, Myocardial Ischemia, Chest Pain, Qualitative Research

## Abstract

**Background:**

Chest pain is a frequent consultation issue in primary care, with coronary artery disease (CAD) being a serious potential cause. Primary care physicians (PCPs) assess the probability for CAD and refer patients to secondary care if necessary. Our aim was to explore PCPs’ referral decisions, and to investigate determinants which influenced those decisions.

**Methods:**

PCPs working in Hesse, Germany, were interviewed in a qualitative study. We used ‘stimulated recall’ with participants to discuss patients with suspected CAD. With a sample size of 26 cases from nine practices we reached inductive thematic saturation. Interviews were audio-recorded, transcribed verbatim and analyzed by inductive-deductive thematic content analysis. For the final interpretation of the material, we used the concept of decision thresholds proposed by Pauker and Kassirer.

**Results:**

PCPs reflected on their decisions for or against a referral. Aside from patient characteristics determining disease probability, we identified general factors which can be understood as influencing the referral threshold. These factors relate to the practice environment, to PCPs themselves and to non-diagnostic patient characteristics. Proximity of specialist practice, relationship with specialist colleagues, and trust played a role. PCPs sometimes felt that invasive procedures were performed too easily. They tried to steer their patients through the system with the intent to avoid over-treatment. Most PCPs were unaware of guidelines but relied on informal local consensus, largely influenced by specialists. As a result, PCPs gatekeeping role was limited.

**Conclusions:**

We could identify a large number of factors that impact referral for suspected CAD. Several of these factors offer possibilities to improve care at the clinical and system level. The threshold model proposed by Pauker and Kassirer was a useful framework for this kind of data analysis.

**Supplementary Information:**

The online version contains supplementary material available at 10.1186/s12875-023-02064-y.

## Background

In primary care, chest pain is a common symptom with a prevalence of 0.7–2.7% among all patients [1–3]. Underlying diseases are manifold and differ considerably regarding their prognosis. Coronary artery disease (CAD) occurs in 10–15% of patients with chest pain in primary care; 11.1% are due to chronic CAD and 3.6% to acute coronary syndrome (ACS)[1]. Because of its prognostic and management implications, primary care practitioners (PCPs) are required to identify patients with CAD [4, 5]. However, distinguishing CAD from other, mostly benign conditions is difficult due to overlapping symptoms or atypical clinical presentations [6]. Thus, PCPs face the challenge of not missing serious disease on one hand, yet on the other, fulfilling their role as gatekeepers, avoiding unnecessary referrals and protecting patients from over-treatment.

Once CAD is regarded as sufficiently probable, relevant investigations are available only in secondary care, except for indicators of acute myocardial infarction (ECG [electrocardiogram] and point-of-care markers). Thus, referral is usually needed to confirm or exclude CAD with sufficient certainty.

In recent decades, referral from primary to specialized care has been extensively researched. High variability of referral rates, which could be explained by patient factors, has led researchers to consider physicians’ beliefs and emotions as a contributing factor, alongside the communication between providers and patients [7–10].

A framework that has been successfully used to explain physicians’ decision making behavior in general is S. Pauker and J. Kassirer’s ‘threshold approach’ [11] and its modifications [12, 13]. Pauker and Kassirer in their model link disease probabilities and values with behavioral clinical consequences (for further explanation see below). R. Cummins et al. hypothesize that physicians have unique referral thresholds that may explain differences in referral rates [8]. We propose Pauker and Kassirer’s threshold model as an appropriate framework to improve our understanding of patient, provider and context factors impacting on referral decisions for suspected CAD.

Research presented here was part of a larger project investigating use and possible overuse of coronary angiograms and interventions in Germany (the KARDIO-study^1^), funded by the Innovation Fund by the Federal Joint Committee (ID: 01VSF16048). Our aim was to explore PCPs’ referral decisions, in patients with suspected CAD and to investigate determinants influencing those decisions.

## Methods

### Study design

This study was conducted in primary care practices in Hessen, Germany in 2017. We conducted semi-structured interviews to explore PCPs’ diagnostic approach to patients with suspected CAD including referral to specialized care. In order to anchor interviewees’ elaborations in their practice, we developed an interview guide that used the method of ‘stimulated recall’ of diagnostic decisions of previously-seen individual patients. After the opening case descriptions, the second part of our interview-guide consisted of questions on clinical tactics and strategies, collaboration with other providers and the local health care system, as well as the patient perspective (see Additional file 1). The themes of the interview-guide were previously derived from literature research and our experience in collaborative research. The guide was discussed in our multidisciplinary research group and we used the first interview to test prompts and understanding.

### Sampling and recruitment

PCPs were approached via the research practice network of the Department of Family Medicine at the University of Marburg/Germany. We sent postal invitations with information about the study’s aims and procedure to 117 PCPs. We received positive responses by 30 PCPs. Criteria for selecting participants were as follows: geographical variation (urban/rural); balanced gender ratio; variation of professional experience. We contacted the PCPs (*n* = 10) by telephone to schedule the interviews and sent them material for the ‘stimulated recall’. Participants were asked to consecutively document three patients. These were to have symptoms suggestive of CAD within the last two weeks, for whom the PCP was considering a coronary angiogram, but was uncertain about how best to proceed. Case report forms were only intended for use by participants to recall respective patients and consultations. In addition, participants could use their routine clinical documentation.

### Data collection

The semi-structured interviews were conducted by NG, who has extensive experience in conducting qualitative interviews. Our interview guide aimed to create a natural conversational atmosphere [14]. All interviews took place in the PCPs’ offices in a largely undisturbed setting. The interviewer made field notes during and after the encounter. The interviews were digitally recorded, pseudonymized, and transcribed verbatim. Transcribed data were imported into MAXQDA® (version 18.2.0), qualitative data analysis software.

### Qualitative analysis

We based data preparation and qualitative content analysis on Kuckartz’ approach [15]. Two members of the research team (AB, KW), who were doctoral candidates and medical students, initially created a coding tree based on the key questions of our interview guide. By encoding the material independently from each other, they defined new codes that emerged from the material, followed by regular consensus discussions with NG, KS and NDB from the research team as well as the departments Working Group on Qualitative Research. Hereby, we inductively formed an abstracted category system. Additionally, the interviews were summarized on the content level, in order to review the codings in the context of the entire interview. During analysis, we learned, that the diagnostic episode usually ends with PCPs referral decisions and that a decision about coronary angiograms is discussed elsewhere. Thus, we selected categories relevant to the subject of referral using the ‘code-relation browser’ and ‘code overlap function’ of our QDA-Software.

We transferred the inductively identified codes into a matrix and examined each factor regarding its coherency between cases and its influence on referral decision (pro versus contra referral, bidirectional depending on context, neutral). In order to comprehensibly contextualize and present the determinants, we drew on the decision threshold model.

### Application of threshold model

The decision threshold model by Pauker and Kassirer [11] links disease probabilities, values and behavioral clinical consequences. After collecting diagnostic information by taking the patient’s history, performing a physical examination and perhaps laboratory testing, the probability of disease may be so high that no further diagnostic is warranted, but treatment should be started instead. In that case, disease probability is said to have crossed the *therapeutic threshold*.

In applying the threshold model to the decision of whether to refer a patient or not, we have modified Pauker’s and Kassirer’s concept in two ways. First, while the original understanding of the model has been normative (to decide whether to continue diagnostic testing, or instead start treatment) [11], we have used it as an *analytical heuristic* for explaining the behavior of health professionals. Second, we have broadened the applicability of the model to the whole of the diagnostic process, whereas the focus by Pauker and Kassirer was on a single, often risky investigation potentially indicated after history taking, clinical examination, biochemical tests etc. We regard this broader applicability of the model as particularly useful for the primary care setting, because the length and the intensity of the diagnostic process varies considerably depending on the patient’s problem [16, 17].

**Fig. 1:**
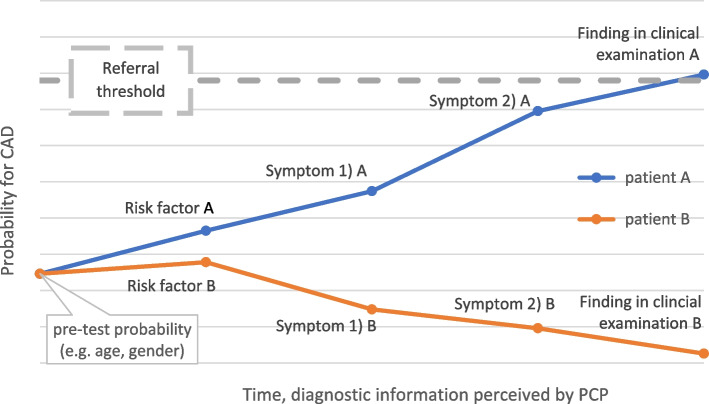
Illustration of threshold model according to Pauker and Kassirer 1980 [11] in the context of suspected CAD in primary care, modified

Figure 1 illustrates the idea of the modified threshold model. The abscissa denotes time and parallels the cumulative information obtained throughout the diagnostic process; the ordinate axis shows the probability for CAD. At the beginning, two patients with chest pain may present equal pre-test probabilities. Individual diagnostic information leads to diverging probabilities for CAD. If the probability for CAD exceeds a certain level (dotted line) the patient is referred for assessment and investigations, in most cases to a cardiologist. We refer to this probability level as the *referral threshold.*

While the patient’s symptoms and signs modify the probability for CAD, we assume that variations in behavioral consequences, such as referrals, can be explained by factors influencing thresholds at the level of the physician or the wider health care system context. Thus, we believe, that the referral threshold is not at a stable probability value but is being modified by individual and more general determinants.

The interviews were conducted in German. For this publication, quotations were translated by the authors. The study is reported in accordance with the guidelines of the consolidated criteria for reporting qualitative research (COREQ, see Additional file 2). The study was approved by the Ethics committee of the University of Marburg in June 2017 (74/17).

## Results

### Sample

We invited 117 PCPs for participation of which 42 responded, and 30 were willing to participate. Of those, we selected 10 PCP (see method section). However, one interview had to be excluded due to technical reasons and reported cases that did not meet our inclusion criteria. Within nine interviews covering 26 reported patients, we achieved an inductive thematic saturation with high redundancy of mentioned themes regarding referral decisions. The interviews lasted between 26 and 62 min (44.8 min on average). The instructions regarding stimulated recall were largely followed (more details see Additional file 2: COREQ). Two PCPs reported fewer than three cases, in one case the physician was asked for a second opinion but did not make the final decision himself. Characteristics of interviewed PCPs are given in Table 1. The reported patients were aged between 40 and 90 (mean: 68 years), 35% were female. Additional file 3 presents demographic data of patient cases.

**Table 1:** Characteristics of the included PCPs

PCPs’ characteristics	Number of PCPs
Gender	
Male	6
Female	3
Age group	
< 50 y	1
50 – 59 y	6
60 – 69 y	2
Experience (years since establishment)	
< 10 y	1
10—14 y	1
15—19 y	3
> 20 y	4
Type of practice	
Single-handed	4
Group practice	5
Area of practice	
Large city: > 100 000 inhabitants	1
Medium-sized town: 20 000–100 000 inhabitants	1
Small-sized town: 5000–20 000 inhabitants	6
Rural community: > 5000 inhabitants	1
Availability of exercise ECG	
Conducted in own practice	2
Not available in own practice	7

### Factors influencing referral thresholds

**Fig. 2:**
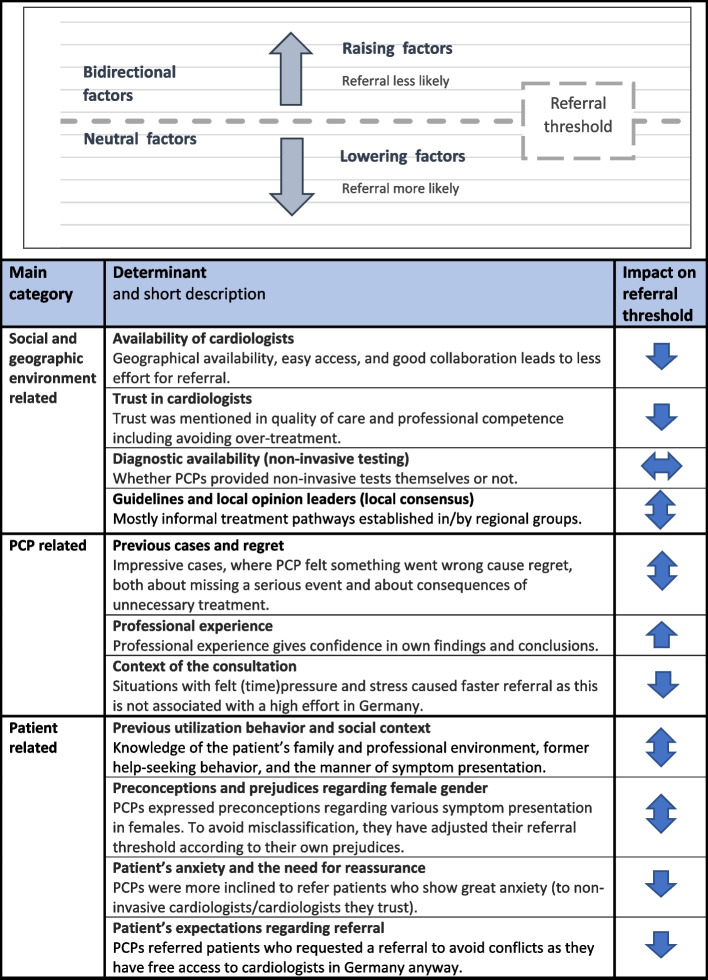
Overview of threshold-determining factors. The graphical part on the upper shows the referral threshold as a grey dashed line, the large arrows symbolise the direction in which the threshold is shifted. On the lower side, the determining factors are listed in tabular form according to their main category, their impact on referral threshold is shown by arrows. The results shown here represent inductively derived factors of PCPs (data and not theory-driven approach)

In total, we identified 11 factors associated with PCPs’ referral thresholds. They relate to three main categories: social and geographic environment, practitioner-related and patient-related factors, respectively. We sought to determine whether these factors raised or lowered the referral threshold (see Fig. 2).

#### Social and geographic environment

The decision of the PCP whether to refer a patient to the cardiologist is influenced by the immediate and wider regional environment in which the consultation takes place.

##### Availability of cardiologists

In Germany, a large number of community cardiologists provide ambulatory care. Some of these own or hire cardiac catheter laboratories for non-acute cases. Patients with statutory health insurance, which is the most common healthcare arrangement in Germany, have direct access to cardiology practices. However, the majority use the route of a referral by their PCP.

Access (and thus a lower threshold for referral) depends on the number and proximity of cardiologists in the region, but also on previously established collegial relationships. In the latter case, contact is made quickly resulting in advice or speedy appointments.

*“So, I work [...] with both big [cardiology] practices here, so it is a very good and trusting relationship, and they [the cardiologists] react immediately if we have the impression that there is an urgent need for action. So, I get an appointment immediately [...]. We also have personal phone numbers of our colleagues and can reach them directly.” [P-04, 47]*

Conversely, the referral threshold is higher if the cardiologist’s practice is far away from the PCP and/or the patient’s home. Moreover, lacking a personal knowledge of the cardiologist, the effort seems to be higher than otherwise.

*“The barrier is greater for me. I do not know my colleague [cardiologist], just over the phone. Calling him, until I get to him, through practice staff, takes quite a while. It costs me time, it costs me effort. I do not have a phone number of anyone to make things shorter. That means that there is a greater barrier. For me, for the patients. They must get there, [...] they must ask their children to drive, they must take time off, and so on." [P-08, 154]*

##### Trust in cardiologists

In regions where several community cardiologists are available, PCPs choose partners for their informal network based on respectful communication, reliable discharge letters and absence of over-investigation and over-treatment.

Interestingly, we found that some PCPs differentiate between sending patients to an interventional or a non-interventional cardiologist. PCPs choose referral to a non-interventional cardiologist if they regard the probability for coronary stenosis as low, and invasive procedures as probably not indicated. They still refer for their own or the patient’s reassurance, or because they prioritize treatment problems other than coronary stenosis.

P-07 reported a 55-year-old patient with attacks of palpitations over two weeks, whom he referred to a non-interventional cardiologist for non-invasive testing. Commenting on his decision, the PCP said:

*“I advised him [the patient] that he should see a cardiologist, I think you have to do an exercise ECG, just because of this presentation. Consequently, he also needs a cardiac ultrasound [...]Basically, he goes to the [non-interventional] cardiologist, because I do not really think he needs a coronary angiogram. “[P-07, 8]*

P-07 further reported a patient with low probability for CAD, but who was desperate for a cardiological opinion:

*“If he keeps besieging me and says he wants that, I will send him to the cardiologist and say, then the cardiologist has to struggle with him. Then, I do not send him to the cardiac catheter, I send him to the [non-interventional] cardiologist.” [P-07, 116]*

Patients with a high probability for CAD for whom the PCP assumes further testing and coronary angiography to be beneficial are referred to interventional cardiologists. P-08 talks about a 73-year-old patient with known CAD and pressure on his chest identical to earlier origin of CAD pain:

*“And then I *picked* up the phone directly, […], told myself that it [the artery] might have been clogged again, […] and then registered him [for the coronary angiography].” [P-08, 78ff.]*

##### Diagnostic availability

The range of available technical procedures is limited in primary care practice. Some primary care physicians offer noninvasive testing such as exercise ECG or echocardiography. Practitioners faced with the possibility of testing mentioned that they would refer if stress testing was positive, but most patients were either unsuitable or returned a normal test result. The exercise ECG thus had a very limited role. They expected the cardiologist to perform non-invasive investigations anyway. P-03 puts it:

“I knew that the colleague [cardiologist] would do an ergometry anyway. I can save myself the effort.” [P-03, 21]

The availability of non-invasive testing in primary care practice thus does not seem to have an impact on the referral threshold.

##### Guidelines and local opinion leaders

Some PCPs mentioned local standards regarding referral for suspected CAD. Interestingly, national or international (European) guidelines were not mentioned. Behaviors and beliefs seemed to be influenced by locally-negotiated rules that arise from the interaction with local opinion leaders such as hospital specialists.

*“The hospital has formed a group of established practitioners and their chief physicians who meet once a quarter in the hospital and talk about the problems of the established with the hospital. […] Such things are also discussed, including procedures [regarding CAD], and we have - the hospital issues so-called newsletters, where recommendations are suggested for the established colleagues in the whole district, yes. And every established colleague gets that, all PCPs, this is such a guideline, which concerns the current diseases.” [P-05, 38ff.]*

In most cases, however, local consensus was implicit.

#### Practitioner-related factors

##### Adjustment of thresholds in the light of previous cases

PCPs mentioned remarkable cases in which the real outcome differed from the expected. P-05 explained that he had become very cautious about patients with chest pain because of the following incident. A nurse, whom he was acquainted with, presented with back pain and asked to be referred to an orthopedic specialist. The PCP ordered an ECG and found an ST elevation as a sign of myocardial infarction. The patient had to be resuscitated in the ambulance and eventually died of cardiac arrest. He formulated the consequences he drew from this as follows:

*“That affected me very deeply. I must say. [...] Because I did not expect it, yes. [...] So that is why I am particularly careful with women, if they have retrosternal pain and also with nausea and back pain, then I always think of the worst case. […] I no longer take any risks, better to admit once too often [...]. And even if I have the slightest suspicion, they go to the hospital.” [16ff.]*

Interestingly, although this PCP did not commit an error, the case was so impressive that he decided to operate with a lower referral threshold in the future. At the same time, PCPs also reported feelings of regret after wrongly-referred patients suffered harm from invasive procedures.

P-08 reports of an 82-year-old woman who could no longer climb the stairs in her three-story house without having to stop twice because of angina and shortness of breath. Coronary angiography showed mitral valve disease and generalized CAD. The patient underwent coronary artery bypass grafting. Post-operatively, she developed heart failure and a severe MRSA infection. She died six months later. P-08 commented:

*“She would be alive for sure if she hadn't been privately insured and if she hadn't gone to the cardiologist. She might need four breaks to get up those stairs now, but she would still be alive. And that is the reason for my attitude: wait and see first. I'm not a fan of too many technical investigations." [P-08, 138]*

In cases of missed or nearly missed CAD, the consequences anticipated by PCPs amounted to lowered threshold for referral. After wrongly assuming CAD and referring patients for coronary angiographies and/or hospitalization, PCPs would rather raise their referral threshold in subsequent cases.

##### Professional experience

In addition to individual cases with high impact, long-term clinical experience seems to be relevant. A widely-held view amongst interviewees was that experienced doctors assess their patients differently from novices. Experience gives PCPs confidence in their own findings and conclusions. As a result, they use conservative strategies such as watchful waiting more often than earlier in their career. They also rely more heavily on history and physical signs than investigations. For example, one interviewee said:

*“But these are the young colleagues who have faith in laboratories and technology [...]. Especially when they come out of the hospital [training]. And then, as, yes, experienced colleagues, we can say: Take it easy. If the patients are doing as they always are, there is no reason to send them to the hospital for catheters or because of decompensated diabetes. There is no reason, we just have to take a close look at our patients." [P-08, 94]*

##### Context of the consultation

Another aspect that can affect the PCPs’ inclination to refer a patient are the conditions around the consultation. Feelings of being time pressured, stressed, or annoyed seem to have an impact on referral thresholds. P-08 mentions Monday morning stress and a crowded waiting room leading to referrals which would otherwise not have happened:

*“If I were to evaluate that statistically, I probably wrote most of the referrals to cardiac catheter on Monday morning. Because the practices are usually all jam-packed. And because I have less time and less desire to deal that intensively with patients, whether they really have a hard indication for a catheter. […] So, when the waiting room is full outside, the referrals are in there. That goes in no time. On Friday morning I talk to the patient for half an hour and then we decide whether it makes sense or not.” [P-08, 114ff.]*

#### Patient-related factors

While symptoms and signs help determine individual CAD probability, there are other patient characteristics which impact the referral decision, such as former help-seeking behavior, gender and patient expectations.

##### Previous utilization behavior and social context

Knowledge of the patient’s family and professional environment, her former help-seeking behavior, and the manner of symptom presentation influence the doctor’s inclination to refer. The PCPs set the referral threshold lower for patients who attend rarely (“coming late”) or patients of low social status. They are reluctant to refer patients who consult frequently (especially for minor problems), patients with panic attacks and other mental health problems. For example, P-09 said:

*“For how long I have known him, whether this is a patient who always comes for all sorts of things or whether someone comes rarely, that matters. So if someone is not here that often and then comes with such complaints, then […] the warning lights are more likely to go off.” [P-09, 40]*

Knowing patients from previous consultations helps to interpret the presentation. A patient who is more likely to dissimulate may then be treated differently than a patient describing complaints in a dramatizing fashion. In new patients, however, this kind of background knowledge is missing. They are treated with “more caution”, i.e. additional investigations. P-07 described a patient he treated while covering for a neighboring practice:

*“In the end I gave her painkillers, told her she had nothing heart-related, and wrote down for me to do an exercise ECG. But more from the point of view that I do not know her, that I did not want to do anything wrong to her, that one does not want to overlook anything.” [P-07, 70]*

Another characteristic mentioned is local prominence. Especially in rural communities, regret is anticipated if a prominent person such as the local mayor presents symptoms, as the following citation illustrates:

*“If I had missed something there [with the patient who is the local mayor], I would not have been able to go out on the street. [...] Well, there are patients who expect a bit more from me because they are in public. Whether this is our pastor or a local mayor here. Yes, they are more likely to be sent to the catheter than I might have done for myself or my father.“ [P-08, 26ff.]*

Again, being cautious results in lower thresholds for investigations and referral.

##### Preconceptions and prejudices regarding female gender

PCPs expressed preconceptions regarding various symptom presentation in females. Some PCPs stated that in their view, women had a lower threshold for using primary care services and often expressed emotional problems through physical symptoms. Thus, they felt that referral thresholds should be higher in these cases. For example, one interviewee said:

*"If she [the patient] has had an inconspicuous exercise ECG, that happens occasionally, especially with the fifty-year-old female patients [...], then they get the diagnosis of somatization disorder, meaning psychosomatic, and they never go to the cardiologist or get a catheter […]. Such a female clientele. […] They are usually more sensitive." [P-08, 26f, 100f.]*

However, other interviewees mentioned they would be particularly careful with female patients and possible CAD, as the following citation illustrates:

*P-04: “I referred her[...].” I: “What led you to this decision?” P-04: “Yes, the pre-existing illness [diabetes mellitus II] and her non-compliance [...]. And of course, the fact that the symptoms of heart attacks in women are often very mild or not so typical; so you always have to be more careful.” [P-04, 25ff.]*

Here, the possibility of an atypical symptom presentation in women with CAD and the will to avoid misclassification leads to a lowered referral threshold.

In conclusion, the expectation and subjective perception of gender-specific symptom presentation can have an impact on the individual referral threshold, but in differing directions depending on the PCP’s own preconceptions and prejudices.

##### Patient’s anxiety and the need for reassurance

Many of the interviewed practitioners indicated that they were more inclined to refer if the patient showed great anxiety, and they felt the need to provide reassurance. For example, P-03 says:

*“So it is also a reassurance for the patient if she is sent to the cardiologist. That means that maybe there does not have to be anything, but she knows that she can always have the referral to the cardiologist, and that ultimately gives her a piece of security.” [P-03, 96]*

##### Patient’s referral expectation

Some interviewees reported that they occasionally refer patients, for whom they consider a referral unnecessary, but who still request a referral or even a coronary angiogram. In these cases, PCPs tend to comply with the request and issue the referral. They thus avoid a conflict with the patient. They regard this conflict as not worthwhile because patients in Germany have free access to cardiologists. For example, P-01 said:

“So, if a patient exerts pressure, if he says, "I want to see a cardiologist", of course, I do not start discussing. Well, I would refer him, I would say "I don't think you have anything, but then go".“ [P-01, 116]

If reassurance is the main reason, referral to invasive cardiologists is usually avoided and noninvasive chosen instead (see 1.2 ‘Trust in cardiologists’).

## Discussion

### Key findings

By recalling difficult decisions in previous patients with suspected CAD, PCPs provide detailed accounts of their reasoning for or against a referral. Apart from patient characteristics determining disease probability, we have identified more general factors which can be understood as influencing the referral threshold. These factors relate to the practice environment, to PCPs themselves and to non-diagnostic patient characteristics.

### Published literature and implications of findings

It is highly plausible and in keeping with the literature that the proximity of specialized practice and relationships between physicians impacts on referral thresholds [18, 19]. What is remarkable here is the relevance of trust, and potential conflicts of interest. Apparently, referring PCPs have experienced cardiologists who differ regarding their tendency to perform invasive procedures. If they want a cardiological opinion on a patient but regard CAD-probability as low, they seem to fear some cardiologists will perform inappropriate testing and procedures on their patients. In these cases, they would rather refer to cardiologists who do not provide coronary angiography themselves, presumably to avoid biased decisions resulting from cardiologists’ conflict of interest.

It is well known that local norms are at least as important as explicit standards to understanding provider behavior, such as clinical practice guidelines, which are usually developed and published at a national or international level [20]. Physicians interviewed in our study were mostly unaware of the national and the European guidelines, which would have suggested a different approach to dealing with suspected CAD. Local specialists were apparently successful in shaping local medical opinion.

Feedback from previous decisions and their outcomes have been shown to influence future behavior, which can be framed as a change in decision thresholds [21]. Our findings underscore the relevance and high potential for regret associated with suspected CAD, but also the utility of the threshold concept to understand provider behavior.

Study participants felt that with longer clinical experience they were more in a position to rely on the history and non-invasive findings. This suggests that training and active guideline implementation can improve PCPs future referral behavior for patients with suspected CAD.

PCPs under time or productivity pressure are at risk of providing low-quality care. That study participants refer more often during busy clinics is in keeping with the literature [22–27]. In addition, these referrals are presumably accompanied by sparse information on the patient. In Germany, in order to refer a patient, only a single form with minimal space for information is required. For referrals regarded as urgent, PCPs often call the specialist by phone. Against a background of increasing numbers of cardiologists providing direct access, an easy referral procedure results in PCPs having only a weak gatekeeper role.

We distinguished symptoms and signs associated with disease probabilities from patient characteristics assumed to influence referral thresholds. In a previous study we could show that PCPs indeed form an opinion about their patients’ utilization thresholds and tailor their investigations accordingly [28]. Our current investigation shows that similar considerations apply to patients with suspected CAD and possible referral.

Comments on gender, in particular the presentation of CAD in women appearing to be different from men, illustrate the dilemma frontline practitioners must face [29–31]. If they want to draw consequences from the suggestion that CAD presentation in women differs from men, in the absence of specific clinical criteria, they have to lower their threshold for assuming the possibility of CAD and refer accordingly. This, however, exposes women to potential over-investigation and overtreatment [32].

### Strengths and limitations

Our study included nine practitioners from different types of practice, and both urban and rural practices were represented. Regardless of the small sample size, we achieved a high variation regarding demographic characteristics, such as age and gender. By recruiting members of a university research network, we probably have selected highly motivated and reflecting PCPs [33]. For this study, we had to rely on selected reports of provider behavior, instead of observed or documented actions. Although we tried to establish a trustworthy relationship with participants, social desirability bias cannot be excluded. The stimulated recall method proved to be productive in making meaningful reflection on included cases possible. We cannot exclude that PCPs have chosen impressive cases instead of consecutive ones. However, as our interviews revealed rich data to explain PCPs behaviors, we assume our study nevertheless valid and relevant. We used Pauker’s and Kassirer’s threshold model to analyze PCPs’ reflections on their referral decisions. While the model has been used for quantifying decision thresholds on the basis of case vignettes [34], we took it as a heuristic for qualitative data analysis. Its main advantage has been to help us separate two components of the referral decision: 1) diagnostic features presented by the individual patient; 2) other, more general factors beyond the individual clinical picture which were also important. Overall, these components could be distinguished clearly. One exception was patients’ anxiety and subsequent need for reassurance. This is a characteristic that affects the PCPs referral threshold, but is also diagnostic for CAD in primary care [3, 35]. In this case, we interpreted the patient’s evaluation of her symptoms as influencing the decision threshold.

Readers should be aware that our results are based on a qualitative study of a comparatively small sample recruited in several regions in a state of Germany. Confirmation in future studies is desirable.

### Policy implications

Our qualitative data can neither prove nor refute the hypothesis that invasive coronary procedures are performed too often in Germany [36]. However, PCPs express beliefs and behave in a way that seems to indicate an oversupply of invasive cardiologists in their region, a risk for over-investigation, over-treatment and, as a consequence, the need to protect their patients. This interpretation is supported by international comparisons in which Germany has top positions for coronary angiograms, PCI (percutaneous coronary intervention) and the number of interventional cardiologists [37–40].

Our findings suggest that the composition of the medical workforce forms a barrier against the implementation of evidence-based practice guidelines, undermine trust between physicians and prevent PCPs from assuming a stronger gatekeeper role.

The imbalance in the medical workforce and excessive procedures performed together with our findings are a case for a tighter regulation of the number of specialists in Germany, institutional support for primary care gatekeeping and the implementation of primary care guidelines. In order to incorporate patients’ preferences, patient information material and decision aids are available and deserve more intense implementation. This is particularly relevant since thresholds ultimately depend on values, as shown by Pauker and Kassirer [11].

The results of our study apply to health care systems with insufficient control of the specialist work force and weak primary care gatekeeping.

## Conclusions

In this study, we demonstrate a large number of factors influencing the referral decision of PCPs in patients with suspected CAD. Some of them indicate an erosion of trust resulting from an oversupply of specialists, leading to over investigation and over treatment. PCPs feel the need to protect their patients. However, they are not aware of current guidelines, nor are they in a position to act as effective gatekeepers. The threshold model proposed by Pauker and Kassirer served us well as a framework for qualitative data analysis.

## Supplementary Information

**Additional file 1.**

**Additional file 2.**

**Additional file 3.**

## Data Availability

The datasets analyzed during the current study are not publicly available, because they contain highly sensitive information. Requests to access the data in German can be sent to the corresponding author.

## Abbreviations

CAD: Coronary artery disease
PCI: Percutaneous coronary intervention
CA: Coronary angiogram, coronary angiography
ECG: Electrocardiogram
PCP: Primary Care Physician
P: Participant
I: Interviewer

## Footnotes

1 KARDIO-Study: Three components: A) Identification of regional variations of invasive coronary angiography use in Germany (routine-data analysis); B) Identification of differences of factors influencing the rate of invasive coronary angiography in high- vs. low-use regions (qualitative study); C) Implementation of local interdisciplinary clinical pathways (intervention study).
