# Supplementary material for: Determinants of referral for suspected coronary artery disease: a qualitative study based on decision thresholds
Source: BMC Prim Care. 2023 May 2;24:110. doi: 10.1186/s12875-023-02064-y (PMC10152784; doi:10.1186/s12875-023-02064-y)
Supplement: Supplementary file 1 — Additional file 1. [file 12875_2023_2064_MOESM1_ESM.docx]

**Additional file 1:** Interview guide for semi-structured interviews with PCPs

| **Main Questions** | **Follow-up questions & possible question content** |
| --- | --- |
| **Stimulated Recall:**  You have documented patients who either have symptoms suggestive of CAD or who are known to have CAD and in whom one can consider a diagnostic cardiac catherterisation. **Do you have such a case that you can describe to us?** | |
| - If no: vignette | |
| - If yes: ask later if there is also a complex case | |
| Case usual / unusual? | |
| What kind of patient was that? | |
| What was the procedure? (history / basic diagnostics / non-invasive examinations (functional tests) / referral decision?)  What was the next step? | |
| Was the decision easy or difficult? | |
| What was easy / difficult? | |
| - If simple: is that generally the case? | |
| - When is it difficult to decide? | |
| For whom is the decision difficult? (Practitioner - Patient) | |
| Which point was decisive for the decision in the end? | |
| To what extent do guidelines help you in such a situation? | |
| **Influencing factors** | |
| (Emotion) Objective findings are not always clearly groundbreaking. In such situations influence of gut feeling, intuition or similar? | |
| Characteristics / features of patients, their wishes or current situations, with influence?  Men / women / age? | |
| Does how the patient appeared / how they described their concerns have an influence? | |
| Influence of context? Situation? | |
| What / which of these aspects is most likely to decide in case of doubt? | |
| **Heuristics** | |
| Have you found a rule of thumb for yourself based on your wealth of experience? | |
| Do you have a typical sequence of steps?  (sequence of actions in the diagnostic process / treatment steps) (e.g. with feedback from/agreements with colleagues etc.) | |
| To which cardiologists do you refer patients? Are there any considerations in this regard? | |
| Are there patients where one typically does / does not do a non-invasive examination? | |
| What criteria must be in place for you to assess things urgently / relaxed? | |
| What do you think such criteria look like for other practitioners? | |
| Cooperation and local structures | |
| What is the practice structure like - exchange in difficult cases? | |
| Is there cooperation with colleagues (other PCPs - cardiologists - clinic) that leads to certain treatment procedures? | |
| What is the significance of nuclear medicine examinations?  What is the significance of the stress ECG for you? | |
| As far as the professional environment is concerned - are there expectations or social norms for certain procedures (unspoken tenor)?  Where do you see yourself? | |
| Is the cardiologists' threshold for cardiac catheterisation too low or high? (too early or too late?) | |
| **Incentives** | |
| Do cardiologists have incentives for cardiac catheterisation these days through the fee prescription structure?  How much do you think this matters (or not)? | |
| If you could change something:  Do you see any undesirable developments or need for change in the care system? | |
