# Supplementary material for: Determinants of referral for suspected coronary artery disease: a qualitative study based on decision thresholds
Source: BMC Prim Care. 2023 May 2;24:110. doi: 10.1186/s12875-023-02064-y (PMC10152784; doi:10.1186/s12875-023-02064-y)
Supplement: Supplementary file 2 — Additional file 2. [file 12875_2023_2064_MOESM2_ESM.docx]

**Additional file 2:** Consolidated criteria for reporting qualitative studies (COREQ)

**Manuscript: Determinants of referral for suspected coronary artery disease: A qualitative study based on decision thresholds**

**Consolidated criteria for reporting qualitative studies (COREQ): 32-item checklist**

(Tong A, Sainsbury P, Craig J. Consolidated criteria for reporting qualitative research (COREQ): a 32-item checklist for interviews and focus groups. *International Journal for Quality in Health Care*. 2007. Volume 19, Number 6: pp. 349 – 357)

| **No. Item** | **Guide questions/description** | | | **Reported on Page #** |
| --- | --- | --- | --- | --- |
| **Domain 1: Research team and reﬂexivity** | | | | |
| *Personal Characteristics* | | | | |
| 1. Inter viewer/facilitator | Which author/s conducted the interview or focus group? | | | Page 5; line 13. Interviews were conducted by NG. |
| 2. Credentials | What were the researcher’s credentials? E.g. PhD, MD | | | Title Page: MSc, Research Fellow |
| 3. Occupation | What was their occupation at the time of the study? | | | Page 5, line 13-14. NG was employed by University of Marburg (Research Fellow). |
| 4. Gender | Was the researcher male or female? | | | Title Page. Female. |
| 5. Experience and training | What experience or training did the researcher have? | | | NG has a background in public health (M.Sc.) and social psychology (M.Sc.). As a senior health care researcher she has been involved in several projects of qualitative research including competences of self-reflection and giving feedback on interviews. Additionally, she received extensive theoretical and practical training in conducting qualitative interviews. |
| *Relationship with participants* | | | | |
| 6. Relationship established | Was a relationship established prior to study commencement? | | | Page 5; line 2. PCPs were approached via the research practice network of the Department of Family Medicine at the University of Marburg/Germany. Participation in previous studies of the department is not excluded. |
| 7. Participant knowledge of the interviewer | What did the participants know about the researcher? e.g. personal goals, reasons for doing the research | | | Declarations (Page 23). PCPs were briefed on the purpose of the study. They did not receive any personal information about the researcher. |
| 8. Interviewer characteristics | What characteristics were reported about the inter viewer/facilitator? e.g. Bias, assumptions, reasons and interests in the research topic | | | Name of the interviewer, purpose of the study. |
| **Domain 2: study design** | | | | |
| *Theoretical framework* | | | | |
| 9. Methodological orientation and Theory | | What methodological orientation was stated to underpin the study? e.g. grounded theory, discourse analysis, ethnography, phenomenology, content analysis | Page 5; line 21f., page 6; line 19f. We based data preparation and qualitative content analysis on Kuckartz’ approach. We applied a modified version of Pauker’s and Kassirer’s threshold model in order to comprehensibly contextualize and present the determinants. | |
| *Participant selection* | | | | |
| 10. Sampling | | How were participants selected? e.g. purposive, convenience, consecutive, snowball | Page 5; line 2-6. PCPs were approached via the research practice network of the Department of Family Medicine at the University of Marburg/Germany. Criteria for selecting participants were as follows: Geographical variation (urban/rural); balanced gender ratio; variation of professional experience. | |
| 11. Method of approach | | How were participants approached? e.g. face-to-face, telephone, mail, email | Page 5; line 3. We sent postal invitations with information about the study’s aims and procedure to 117 PCPs. | |
| 12. Sample size | | How many participants were in the study? | Page 8; line 17. We invited 117 PCPs for participation of which 42 responded, and 30 were willing to participate. Of those, we selected 10 PCP regarding age, gender and geographic variation. | |
| 13. Non-participation | | How many people refused to participate or dropped out? Reasons? | Page 8; line 18-20, line 23-25. 12 PCPs explicitly refused to take part. One interview had to be excluded due to technical reasons and reported cases that did not meet our inclusion criteria. | |
| *Setting* | | | | |
| 14. Setting of data collection | | Where was the data collected? e.g. home, clinic, workplace | Page 5; line 15. All interviews took place in the PCPs’ offices in a largely undisturbed setting. | |
| 15. Presence of non-participants | | Was anyone else present besides the participants and researchers? | No. PCPs were interviewed individually. | |
| 16. Description of sample | | What are the important characteristics of the sample? e.g. demographic data, date | Page 8 f.; Table 1. Most PCPs were between 50 and 59 years old with > 15 years of work experience and practiced in a small sized town. There was a balanced ratio between those working in a single handed practice and those working in a group practice. 3 PCPs were female, 6 were male. | |
| *Data collection* | | | | |
| 17. Interview guide | | Were questions, prompts, guides provided by the authors? Was it pilot tested? | Page 4; line 13-20; Additional File 1. The interview guide consisted of opening case descriptions (stimulated recall) and questions on clinical tactics and strategies, collaboration with other providers and the local health care system, as well as the patient perspective (see appendix). The themes of the interview-guide were previously derived from literature research and our experience in collaborative research. The guide was discussed in our multidisciplinary research group and we used the first interview to test prompts and understanding. | |
| 18. Repeat interviews | | Were repeat interviews carried out? If yes, how many? | No. | |
| 19. Audio/visual recording | | Did the research use audio or visual recording to collect the data? | Page 5; line 16-17. The interviews were digitally recorded, pseudonymized, and transcribed verbatim. | |
| 20. Field notes | | Were ﬁeld notes made during and/or after the interview or focus group? | Page 5; line 15-16. The interviewer made field notes during and after the encounter. | |
| 21. Duration | | What was the duration of the interviews or focus group? | Page 8; line 22. The interviews lasted between 26 and 62 minutes (44.8 minutes on average). Reasons for a short interview duration were that two PCP reported fewer than three cases, in one case the physician was asked for a second opinion but did not make the final decision himself. | |
| 22. Data saturation | | Was data saturation discussed? | Page 8; line 20-21. Within nine interviews covering 26 reported patients, we achieved an inductive thematic saturation with high redundancy of mentioned themes regarding referral decisions. | |
| 23. Transcripts returned | | Were transcripts returned to participants for comment and/or correction? | No. | |
| **Domain 3: analysis and ﬁndings** | | | | |
| *Data analysis* | | | | |
| 24. Number of data coders | | How many data coders coded the data? | Page 5; line 21. Two members of the research team (AB, KW), who were doctoral candidates and medical students encoded the material independently and completely from each other. NG and KS coded dense passages and where involved in discussions of the material together with NDB. | |
| 25. Description of the coding tree | | Did authors provide a description of the coding tree? | The initial coding tree consisted of the main categories: 1) PCPs diagnostic considerations (e.g. decisive factors, diagnostic uncertainty, professional experience, gut feeling, rule formulation, fear of overlooking something, risk-benefit ratio), decision-making (e.g. timing of the decision, simple/complex decisions, shared decision-making), 2) Referral process and patient factors (e.g. home visit, access to cardiologist, infrastructure, choice of cardiologist), doctor-patient level (e.g. consultation frequency, communication, patient attitude, patient characteristics) and 3) cooperation and structures (e.g. collegial/interdisciplinary, expectation and social pressure), care situation (e.g. education/training, regional care, competition, incentives), guidelines.  Within the analysis of the referral process, we reorganized codes to the three main categories PCP and patient related as well as environment.  The identification of factors out from categories was performed in a second analytic step using matrices. | |
| 26. Derivation of themes | | Were themes identiﬁed in advance or derived from the data? | Page 5; line 23f. Inductive-deductive approach: Two members of the research team (AB, KW) initially created a coding tree based on the key questions of our interview guideline. By encoding the material independently from each other, they defined new codes that emerged from the material, followed by regular consensus discussions with NG, KS and NDB from the research team as well as the departments Working Group on qualitative Research. | |
| 27. Software | | What software, if applicable, was used to manage the data? | Page 5; line 9; line 17-18. MAXQDA® (version 18.2.0), qualitative data analysis software. | |
| 28. Participant checking | | Did participants provide feedback on the ﬁndings? | No. | |
| *Reporting* | | | | |
| 29. Quotations presented | | Were participant quotations presented to illustrate the themes/ﬁndings? Was each quotation identiﬁed? e.g. participant number | Page 10-18. Yes. | |
| 30. Data and ﬁndings consistent | | Was there consistency between the data presented and the ﬁndings? | Page 10-18. Yes. | |
| 31. Clarity of major themes | | Were major themes clearly presented in the ﬁndings? | Page 10-18 and Figure 2. | |
| 32. Clarity of minor themes | | Is there a description of diverse cases or discussion of minor themes? | Page 10-18. Yes. | |
