# Supplementary material for: Determinants of referral for suspected coronary artery disease: a qualitative study based on decision thresholds
Source: BMC Prim Care. 2023 May 2;24:110. doi: 10.1186/s12875-023-02064-y (PMC10152784; doi:10.1186/s12875-023-02064-y)
Supplement: Supplementary file 3 — Additional file 3. [file 12875_2023_2064_MOESM3_ESM.docx]

**Additional File 3:** Demographic Data of reported patient cases

| **Patients’ characteristics** | **Number of patients** |
| --- | --- |
| Gender  Male  Female | 17  9 |
| Age group  < 50 y  60 – 69 y  70 – 79 y  > 80 y  No information | 5  6  6  4  5 |
